# Seminal-Plasma Molecular Biomarkers as a Liquid Biopsy of Testicular Function: Toward AI-Ready Sperm-Retrieval Prediction in Non-Obstructive Azoospermia

**DOI:** 10.3390/ijms27135965

**Published:** 2026-07-02

**Authors:** Aris Kaltsas, Fotios Gasparos, Andreas Koumenis, Marios Stavropoulos, Michael Chrisofos

**Affiliations:** Third Department of Urology, Attikon University Hospital, School of Medicine, National and Kapodistrian University of Athens, 12462 Athens, Greece; akaltsas@med.uoa.gr (A.K.); fotisgaspar@yahoo.com (F.G.); a_koumeni@icloud.com (A.K.); stamarios@med.uoa.gr (M.S.)

**Keywords:** non-obstructive azoospermia, seminal plasma, liquid biopsy, sperm retrieval prediction, microdissection testicular sperm extraction, extracellular vesicles, cell-free and non-coding RNA, TEX101, metabolomics, machine learning

## Abstract

Non-obstructive azoospermia (NOA) is characterized by focal and quantitatively limited spermatogenesis, making preoperative prediction of sperm retrieval difficult. Seminal plasma is a biologically plausible liquid-biopsy compartment because it contains testicular, epididymal and accessory-gland secretions enriched with extracellular vesicles, cell-free nucleic acids, proteins and metabolites. This narrative molecular review examines the mechanisms by which germ-cell-derived molecular cargo reaches the ejaculate and organizes seminal-plasma biomarkers by cargo class and spermatogenic stage. Particular attention is given to extracellular-vesicle non-coding RNAs, cell-free seminal mRNAs, germ-cell-enriched proteins including TEX101 and ECM1, and metabolomic and lipidomic signatures. Although several markers show promising discrimination, most remain discovery-stage, single-center and insufficiently validated. The central argument is that the field should move from isolated biomarker nomination toward locked, stage-mapped multi-analyte panels integrated with clinical and genetic predictors under modern prediction-model standards. Seminal plasma is best viewed not as a ready clinical test, but as a biologically coherent platform for future calibrated, externally validated and artificial-intelligence (AI)-ready sperm-retrieval decision support.

## 1. Introduction

Azoospermia—the complete absence of spermatozoa from the ejaculate—affects approximately 1% of the general male population, whereas it is identified in approximately 10–15% of men evaluated for infertility, a selected population undergoing semen analysis [1,2]. It is broadly classified as obstructive azoospermia (OA), in which spermatogenesis is intact but the excurrent ducts are blocked, and non-obstructive azoospermia (NOA), in which the testis fails to produce mature spermatozoa. Importantly, NOA does not necessarily indicate a complete absence of spermatogenesis; germ-cell development may be absent, severely reduced, focal, or arrested before the formation of mature spermatozoa [1,2]. NOA is the most severe expression of male infertility and the most therapeutically challenging, because the only route to biological fatherhood is the surgical recovery of testicular spermatozoa for use in intracytoplasmic sperm injection (ICSI) [1,3].

Microdissection testicular sperm extraction (micro-TESE), in which the testis is examined under the operating microscope to identify and excise the dilated, opaque tubules most likely to contain active spermatogenesis, is the current standard of care and outperforms conventional and needle-based testicular sperm extraction (TESE) techniques. Nevertheless, sperm are retrieved in only about 40–60% of micro-TESE procedures, and the probability of success depends on etiology, testicular histology, surgical expertise and laboratory processing [2,3]. For nearly half of the patients, therefore, micro-TESE is an invasive, costly and emotionally demanding operation that ends without spermatozoa. These figures are corroborated by meta-analysis: pooled data indicate that micro-TESE retrieves spermatozoa in roughly half of NOA men and outperforms conventional TESE (≈52% vs. 35%) [4], with a comparable pooled retrieval rate of ~44% in Klinefelter syndrome [5].

This uncertainty has driven a decades-long search for a reliable preoperative predictor of sperm retrieval. Conventional clinical and endocrine variables—follicle-stimulating hormone (FSH), luteinizing hormone (LH), testosterone, inhibin B, anti-Müllerian hormone (AMH), testicular volume, patient age and etiology—show inconsistent discrimination when used individually, and the single most informative variable, testicular histopathology, is itself obtained invasively and is subject to sampling error because residual spermatogenesis is patchy [3]. Genetic testing (karyotype, Y-chromosome microdeletions) is decisive in defined subgroups but explains only a minority of idiopathic NOA [1,6]. There is thus a clear need for a non-invasive assay that reports directly on the molecular state of the seminiferous epithelium. This clinical impression is quantitatively supported: a systematic review and meta-analysis found that serum FSH, inhibin B and AMH each provide only limited and heterogeneous discrimination for micro-TESE success, reinforcing the case for biomarkers that report more directly on germ-cell activity [7]; a broad predictor synthesis reached the same conclusion [8].

Seminal plasma is an attractive substrate for such an assay. It is the fluid in which spermatozoa are normally suspended and represents the pooled secretions of the testis, epididymis, seminal vesicles and prostate; it can be obtained non-invasively and repeatedly; and it carries a remarkable molecular diversity of cell-free nucleic acids, extracellular vesicles, proteins and metabolites that may mirror the activity of the testis even when no spermatozoa are present in the ejaculate [9,10]. The conceptual parallel is the circulating “liquid biopsy” used in oncology, adapted to a compartment that is anatomically downstream of, and enriched for, the tissue of interest.

Several narrative and systematic reviews have catalogued candidate seminal-plasma biomarkers or surveyed artificial-intelligence (AI) prediction models for micro-TESE, but they have largely treated the molecular and computational studies in isolation [8,11]. This review takes a deliberately molecular and mechanistic stance. This review first describes the cell biology that allows germ-cell-derived molecules to enter the ejaculate; it then organizes the biomarker evidence by molecular cargo class—non-coding RNAs, cell-free mRNAs, proteins and metabolites—mapping each analyte onto the spermatogenic stage it reflects and its analytical maturity; and finally it examines how these molecular signals might be integrated with clinical and genetic data through machine learning (ML). The central argument is that the field has produced abundant molecular discovery but has not yet translated it into a validated, calibrated, clinically deployable prediction tool, and that closing this “molecule-to-model” gap—rather than nominating ever more candidate markers—is the decisive challenge for the next generation of studies.

### Scope and Literature-Selection Approach

This is a narrative molecular review, not a systematic review or meta-analysis. Human studies and the mechanistic literature relevant to seminal-plasma molecular cargo, extracellular vesicles, cell-free and non-coding RNAs, testis-enriched proteins, metabolomics/lipidomics and prediction-model translation in NOA were prioritized. Key studies were identified from PubMed and Scopus searches, prior systematic and scoping reviews, and citation tracking. The aim was to synthesize biological plausibility, stage mapping and translational readiness rather than to estimate pooled diagnostic accuracy.

## 2. Biological Basis: Spermatogenesis and the Molecular Content of Seminal Plasma

**Figure 1:**
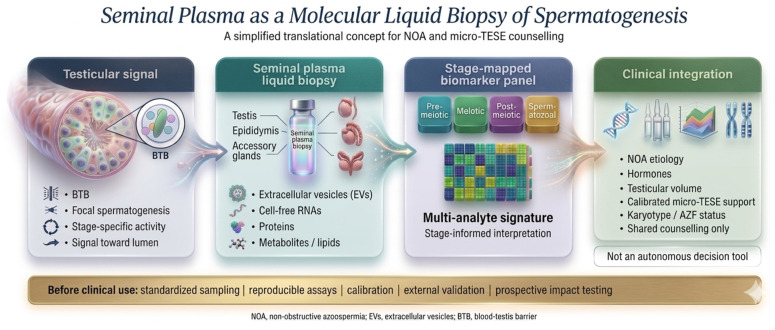
Seminal plasma as a molecular liquid biopsy of spermatogenesis. The schematic illustrates blood–testis-barrier channeling of germ-cell molecules toward the lumen and ejaculate; their release into tubular fluid via extracellular vesicles, apoptotic and residual bodies, and protected cell-free nucleic acids; and the mapping of seminal-plasma cargo classes to spermatogenic stages, assembled toward a candidate prediction model for micro-TESE sperm retrieval. NOA, non-obstructive azoospermia; EVs, extracellular vesicles; BTB, blood–testis barrier.

Seminal plasma can be regarded as a molecular liquid biopsy of spermatogenesis: germ-cell molecules are released into the tubular fluid and directed toward the ejaculate, where they are recoverable as defined cargo classes [12,13]. The underlying biological routes and the resulting cargo classes are detailed in the following subsections and summarized in Figure 1.

### 2.1. Spermatogenesis, Germ-Cell Turnover and the Blood–Testis Barrier

Spermatogenesis is a continuous, spatially ordered process organized into the seminiferous epithelial cycle. Spermatogonial stem cells self-renew and differentiate into spermatogonia, which enter meiosis as spermatocytes; the haploid round spermatids that result then undergo spermiogenesis—nuclear condensation, acrosome formation, flagellar assembly and cytoplasmic remodeling—to become elongated spermatids and, ultimately, spermatozoa. Each step is accompanied by a tightly scheduled, stage-specific transcriptional program, so that the molecular species present at any moment encode information about which spermatogenic stages are active [12].

Two features of this process are central to biomarker biology. First, germ-cell turnover is enormous: a large fraction of differentiating germ cells is eliminated by apoptosis during normal spermatogenesis, and at spermiation elongating spermatids discard cytoplasmic “residual bodies” that are normally phagocytosed and degraded by Sertoli cells [14]. Both apoptotic germ cells and residual bodies are reservoirs of germ-cell RNA and protein that can be liberated into tubular fluid. Second, the seminiferous epithelium is partitioned by the blood–testis barrier (BTB), a junctional complex of claudin-11, occludin and zonula-occludens proteins between adjacent Sertoli cells that creates a privileged adluminal compartment [13]. The BTB restricts paracellular passage of germ-cell molecules into the systemic circulation while allowing luminal contents and Sertoli-cell secretions to move distally through the rete testis and epididymis toward the ejaculate. This compartmentalization has a directional consequence that underpins assay specificity: germ-cell-derived molecules reach seminal plasma preferentially over serum. Seminal plasma can therefore be regarded as a “biopsy” of spermatogenesis, although this directional transit remains biologically plausible rather than directly demonstrated by lineage tracing.

### 2.2. Routes to the Ejaculate: Extracellular Vesicles, Residual Bodies and Cell-Free Nucleic Acids

Germ-cell molecular information reaches the ejaculate through several partly overlapping routes. Extracellular vesicles (EVs) are the best characterized. Their cargo is selectively sorted rather than packaged at random, so that the EV transcriptome and proteome differ systematically from those of the parent cell [15,16]. This selectivity is what allows EV cargo to function as a curated, rather than incidental, molecular signature. In addition to prostate- and epididymis-derived vesicles, the testis itself releases EVs—sometimes termed “testosomes”—that mediate communication among Sertoli cells, germ cells, Leydig cells and testicular macrophages [17,18]. These testicular EVs carry protein and small-RNA cargo and can influence spermatogenesis, and EVs of testicular origin are detectable in seminal plasma, supporting their potential as non-invasive markers of testicular function [17,18].

A second route is the release of cell-free nucleic acids and protein from the high physiological turnover of spermatogenesis. Apoptotic germ cells and incompletely cleared residual bodies plausibly liberate germ-cell RNA, DNA and protein into the tubular lumen, although the demonstration that this specific material transits to seminal plasma remains partly inferential and is an area requiring direct lineage-tracing evidence. A third consideration is molecular stability in a hostile fluid. Seminal plasma is rich in ribonucleases, and naked RNA is rapidly degraded; however, EV encapsulation shields cargo RNA from nuclease attack, and a vesicle-independent pool of microRNA is stabilized through association with Argonaute-2 ribonucleoprotein complexes [19]. These protective mechanisms explain the empirical observation that vesicle-associated and ribonucleoprotein-bound non-coding RNAs are detected far more reproducibly than free RNA, and they justify the methodological emphasis on EV isolation in biomarker workflows. Seminal plasma also carries high ribonuclease activity that rapidly degrades unprotected RNA [20], whereas vesicle- and protein-bound transcripts are considerably more stable [21,22]. The time from ejaculation to processing is therefore a relevant pre-analytical variable, particularly for assays that do not isolate extracellular vesicles, and should be standardized and reported alongside the abstinence interval [23].

### 2.3. Compartmental Heterogeneity and Its Analytical Implications

A crucial caveat is that the seminal EV pool is organ-heterogeneous. Quantitatively it is dominated by prostate-derived prostasomes and epididymis-derived epididymosomes, with a comparatively small testicular contribution; accessory-gland fluids also contribute the bulk of seminal proteins and metabolites. Consequently, a germ-cell-specific signal must be detected against a large background of non-testicular material, which both lowers analytical sensitivity and demands markers with strict germ-cell or testis restriction. This heterogeneity also makes pre-analytical variables—abstinence interval, contribution of accessory-gland fluid, EV isolation method and normalization strategy—major determinants of measured biomarker levels, and it is a principal reason why single-center discovery findings have been difficult to reproduce. These biological realities frame the cargo-class evidence reviewed below and the assay-readiness discussion in Section 5.

Single-cell and spatial transcriptomic studies of NOA testes provide a cellular reference map for interpreting seminal-plasma cargo [24,25]. Germ-cell depletion, Sertoli-cell dysfunction, Leydig-cell alterations, immune activation and extracellular-matrix remodeling may each contribute distinct molecular signals, but only a subset is expected to reach seminal plasma [26].

## 3. Seminal RNA Biomarkers of Spermatogenesis

Seminal RNA biomarkers fall into two complementary classes: regulatory non-coding RNAs and cell-free messenger RNAs. Non-coding RNAs (ncRNAs) are the most extensively studied seminal-plasma cargo class. Many are germ-cell-enriched and decline when spermatogenesis fails, so their abundance in seminal plasma or seminal EVs can act as a surrogate for residual sperm production. Importantly, distinct ncRNA biotypes report on different aspects of germ-cell biology—meiotic progression, transposon silencing, chromatin remodeling and translational control—so that a multi-biotype panel can in principle interrogate several spermatogenic checkpoints simultaneously. The breadth of this regulatory layer is increasingly appreciated: integrated RNA control of the epitranscriptome and three-dimensional genome architecture is now recognized as central to spermatogenesis [27], and small-RNA profiling of azoospermic men has begun to define disease-associated signatures relevant to sperm retrieval [28]. Beyond RNA abundance, RNA modifications are themselves emerging as readouts: an N6-methyladenosine (m6A)-related gene signature has been used to construct a diagnostic prediction model and to define molecular subtypes of NOA, extending the molecular feature space available for prediction [29]. A general caveat applies across these non-coding-RNA studies: most discrimination estimates derive from small, single-center discovery cohorts with internal validation only, so the reported areas under the curve are likely optimistic and would be expected to decline on independent external validation.

To aid a broad readership, several recurring terms are defined here. The area under the receiver operating characteristic curve (AUC) summarizes a model’s discrimination on a 0.5–1.0 scale (0.5 indicating no better than chance and 1.0 perfect separation); a validation AUC refers to this value measured in a held-out or independent cohort rather than in the data used to build the model. The least absolute shrinkage and selection operator (LASSO) is a penalized regression method that selects variables by shrinking less informative coefficients toward zero, thereby reducing overfitting [30]. AI-based (machine-learning) prediction models combine multiple variables to estimate an individual’s probability of an outcome. A prediction threshold is the probability cutoff used to classify patients into clinically meaningful risk groups. The Johnsen score is a semi-quantitative histological score of spermatogenesis in seminiferous tubules, ranging from absence of germ cells to complete spermatogenesis [31]. Throughout, sperm-retrieval success denotes the identification of spermatozoa suitable for ICSI after TESE or micro-TESE.

### 3.1. MicroRNAs

MicroRNAs (miRNAs) are ~22-nucleotide regulators that fine-tune messenger-RNA stability and translation, and several are germ-cell-restricted: the testis-enriched miR-34b/c and miR-449 clusters govern meiotic progression and the histone-to-protamine transition, and their loss produces spermatogenic arrest [32]. In a landmark clinical study, Barceló et al. demonstrated that seminal-exosome miRNAs—most prominently miR-31-5p—discriminate NOA from OA and predict the presence of testicular spermatozoa, with discrimination improving when serum FSH is added to the model [33]. At the cellular level, miR-31-5p controls the proliferation and apoptosis of human spermatogonial stem cells by targeting JAZF1 and cyclin A2 [34]. The same group subsequently validated miR-31-5p as a clinically useful marker and characterized preanalytical influences on its measurement, an unusual and welcome step toward assay robustness [23]. Seminal circulating-miRNA models built by ordered logistic regression have predicted micro-TESE retrieval with high apparent discrimination, the AUC reaching ≈0.93 for retrieval status [35]. Beyond exosomal species, broader semen and even urine miRNA panels have been proposed to flag the presence of spermatogonia in otherwise retrieval-negative testes, although their predictive value awaits validation [36]. Several of these miRNAs participate in regulatory circuits that are also detectable in blood—for example circular-RNA (circRNA)–miRNA axes involving miR-27a and miR-146a-5p—illustrating that a common spermatogenic program leaves correlated footprints across compartments, even if blood-based readouts are inherently less specific than seminal ones [37]. Functionally, miR-27a represses CRISP2 (cysteine-rich secretory protein 2), a protein required for sperm motility and the acrosome reaction, and is overexpressed in NOA, where it may downregulate the histone demethylase KDM3A [38,39]. miR-146a-5p is an inflammation-associated regulator detected in the seminal plasma of infertile men [40].

### 3.2. Long Non-Coding RNAs

Long non-coding RNAs (lncRNAs; >200 nucleotides) regulate transcription, chromatin state and RNA processing, and the testis expresses one of the richest lncRNA repertoires of any organ, much of it stage- and germ-cell-specific. Xie et al. derived a nine-member panel of testis-specific seminal EV-lncRNAs and reported training and validation AUCs of 0.99 and 0.96 for predicting testicular spermatozoa, outperforming serum-hormone models, with a composite score threshold proposed to guide surgery [41]. As the authors and an accompanying commentary noted, the discovery cohort was small and independent external validation is required before clinical adoption. The seminal lncRNA strategy has since diversified. Using weighted gene co-expression network analysis with LASSO selection, Cao et al. identified pairs of seminal extracellular lncRNAs whose relative (ratio-based) expression classified retrieval outcome above a fixed threshold—an approach that is intrinsically robust to normalization because it depends on intra-sample ratios rather than absolute abundance [42]. More recently, a seminal panel of chromatin- and cancer-associated lncRNAs (TUG1, CDKN2B-AS1, H19, Linc-ROR, MALAT1, MIAT, GAS5) showed strong single-marker diagnostic discrimination (e.g., TUG1 AUC 0.94; CDKN2B-AS1 and H19 AUC 0.90), although discrimination for predicting retrieval—as distinct from diagnosing azoospermia—was more modest [43].

### 3.3. Circular RNAs

Circular RNAs are covalently closed transcripts generated by back-splicing; their lack of free ends makes them resistant to exonucleases and therefore unusually stable in biofluids, an advantageous property for a biomarker. Many are abundant in testis and act as microRNA “sponges” or protein scaffolds during spermatogenesis. Ji et al. showed that three testis-derived seminal-plasma circRNAs discriminated micro-TESE outcome in idiopathic NOA, with individual AUCs of 0.89–0.93 and a combined LASSO model AUC of 0.96 [44]. Circular RNAs have also been pursued in less proximal compartments: a six-member testis-derived circRNA panel measured in serum predicted retrieval with very high apparent accuracy (AUC 0.98) [45], a tissue-based nomogram incorporated circ_MGLL [46], and the testicular circRNA hsa_circ_0000116 correlated inversely with Johnsen score and retrieval success [47]. These serum and tissue findings reinforce circRNA biology but are less aligned with a non-invasive seminal-plasma strategy and are best regarded as adjacent evidence.

### 3.4. PIWI-Interacting RNAs

PIWI-interacting RNAs (piRNAs) are ~24–32-nucleotide small RNAs bound by PIWI-clade Argonaute proteins (PIWIL1–4) that silence transposable elements and participate in the histone-to-protamine transition during spermiogenesis; in humans, mutations affecting the piRNA pathway cause azoospermia, underscoring its essentiality for sperm production [48]. Because pachytene piRNAs are produced specifically by meiotic and post-meiotic germ cells, their seminal abundance is mechanistically tied to the presence of those cells. A seminal EV-piRNA logistic model based on pir-61927 distinguished retrieval outcomes (training/validation AUC 0.82/0.83) with favorable calibration [49]. To date, piRNA evidence is stronger for classifying azoospermia origin than for predicting retrieval, and panels remain small and single-center.

### 3.5. tRNA-Derived Fragments

tRNA-derived fragments (tRFs) and tRNA-derived small RNAs (tsRNAs) are produced by regulated cleavage of mature or precursor tRNAs—often by the ribonuclease angiogenin under stress—and modulate translation and gene silencing; notably, sperm acquire tRFs during epididymal transit, where they contribute to paternal epigenetic inheritance. In a two-stage case–control study, seminal EV tRF-Val-AAC-010 and tRF-Pro-AGG-003 distinguished azoospermia origin (both AUC ≈ 0.96), and tRF-Val-AAC-010 predicted micro-TESE sperm presence (AUC 0.89; sensitivity 72%, specificity 91%) [50]. A parallel line of work in circulating blood/plasma exosomes identified tRF-Gly-GCC-002 and tRF-Glu-CTC-005 as retrieval-associated fragments (AUC 0.92 and 0.95) integrated into a nomogram [51]. As for circRNAs, the seminal compartment is mechanistically preferable to blood for a spermatogenesis-specific readout, but the cross-compartment convergence of tRF signals supports their biological relevance.

### 3.6. Cell-Free Seminal mRNAs and Germ-Cell-Specific Transcripts

Cell-free seminal mRNA (cfs-mRNA) is present in the ejaculate at high concentration and with surprising stability, in part because it is protected within extracellular vesicles and ribonucleoprotein complexes; it carries tissue-specific transcripts and therefore functions as a transcriptional liquid biopsy of the male reproductive tract [52]. Its diagnostic power derives directly from the stage-specific timing of spermatogenic gene expression. Pre- and early-meiotic transcripts mark spermatogonia and spermatocytes—*DDX4* (VASA) and *DAZ*/*DAZL* are expressed in pre-meiotic germ cells, and *ESX1* (ESX homeobox 1) marks pre- and post-meiotic germ cells—whereas strictly post-meiotic, haploid transcripts (protamines *PRM1*/*PRM2*, transition proteins *TNP1*/*TNP2*, *SPEM1*, *ZMYND15*, *BOLL*) appear only after meiosis is complete. The detection of haploid transcripts therefore reports “completion of meiosis” and is mechanistically linked to the availability of retrievable spermatozoa, whereas detection of only pre-meiotic transcripts suggests arrest.

The molecular logic that orders these transcripts is itself informative. *ZMYND15* (zinc-finger MYND-type containing 15) is a spermatid-specific transcriptional repressor that temporally gates the onset of a cohort of haploid genes including *PRM1*, *TNP1* and *SPEM1*; its function provides a mechanistic rationale for using these transcripts as ordered stage markers rather than as isolated biomarkers [53]. Clinically, seminal *ESX1* mRNA was detectable in roughly four-fifths of NOA men and predicted sperm retrieval with about 84% sensitivity, although a non-trivial false-negative rate limits its value as a stand-alone rule-out test [54,55]. Hashemi et al. found that the post-meiotic transcripts *ZMYND15*, *TNP1* and *PRM1* (together with *ESX1*) were significantly reduced in non-obstructive azoospermia and showed predictive value for sperm retrieval by receiver operating characteristic (ROC) analysis [56]. Multi-stage cfs-mRNA screens have nominated additional germ-cell candidates—*BOLL*, *AKAP1*, *TCP11* and *SETX*—for detecting completion of meiosis [57].

Several independent observations reinforce this transcript-based strategy. Cell-free seminal *DDX4*/VASA mRNA is depleted in Sertoli-cell-only syndrome and thereby helps separate the most retrieval-unfavorable phenotype from other NOA subtypes [52]. At the tissue level, protamine-1 (*PRM1*) transcript abundance in single testicular spermatids was significantly lower in men with severely impaired spermatogenesis and correlated with post-ICSI pregnancy outcome [58]. Bridging the cellular and molecular views, direct cytological quantification of seminal immature germ cells—combined with mean testicular volume and Johnsen score—produced composite predictive models with AUCs up to ~0.87, indicating that the germ cells whose transcripts are measured can also be enumerated directly in the ejaculate [59]. The individual seminal RNA biomarkers discussed in this section are summarized in Table 1.

## 4. Seminal-Plasma Proteins and Metabolites

### 4.1. The TEX101–ECM1 Protein Pair: The Most Translationally Mature Markers

The best-developed seminal-plasma biomarkers are the germ-cell protein TEX101 (testis-expressed protein 101) and the epididymal protein ECM1 (extracellular matrix protein 1). TEX101 is a glycosylphosphatidylinositol-anchored cell-surface glycoprotein expressed by spermatocytes and spermatids and shed into seminal plasma, so its concentration is a quantitative readout of late spermatogenesis; ECM1, by contrast, reflects epididymal/ductal patency. The pair therefore separates a production problem (NOA) from a transport problem (OA): combined measurement distinguished NOA from OA with 81% sensitivity at 100% specificity, and a TEX101 threshold of ≥0.6 ng/mL predicted sperm or spermatid retrieval with 73% sensitivity and 64% specificity in a preclinical evaluation of 805 samples using a native-protein enzyme-linked immunosorbent assay (ELISA) developed through immunocapture-selected-reaction-monitoring assay cascades [60,61]. The maturity of this pair—from mass-spectrometric discovery, through targeted verification, to a standardized immunoassay—remains the exemplar of how a seminal-plasma biomarker should be translated, and undetectable seminal TEX101 has been proposed as one of the strongest non-invasive predictors of micro-TESE failure in karyotypically normal NOA.

### 4.2. Proteomic Panels and Germ-Cell-Enriched Proteins

Unbiased proteomics has broadened the protein panel beyond TEX101/ECM1. Label-free liquid chromatography–tandem mass spectrometry (LC-MS/MS) of seminal plasma from men with and without retrievable sperm has nominated germ-cell- and testis-restricted proteins—the spermatogenic glycolytic enzymes LDHC (lactate dehydrogenase C) and PGK2 (phosphoglycerate kinase 2), the metallopeptidase DPEP3 (dipeptidase 3), and the germ-cell chaperones HSPA2 and HSPA4L (heat shock protein family A members 2 and 4-like)—as candidate markers, with Western-blot confirmation of reduced LDHC and HSPA2 in NOA and Sertoli-cell-only phenotypes [62]. Independent ELISA-based studies converge on the same biology: seminal PGK2 and acrosin (ACR) were significantly higher in NOA men with successful retrieval, with proposed cutoffs (PGK2 136.3 pg/mL; ACR 21.75 mIU/mL) intended to spare patients futile surgery [63], and comparative LC-MS/MS coupled with conventional assays nominated galectin-3-binding protein (LGALS3BP) as a predictor of successful extraction [64]. Other secreted proteins capture adjacent biology: seminal galectin-1 correlates inversely with Johnsen score (diagnostic AUC 0.86) [65]; the anti-apoptotic protein survivin declines with spermatogenic failure and has been proposed as a seminal marker of sperm retrieval [11,66]; and seminal angiotensin II is lowest in Sertoli-cell-only histology [67]. Sertoli-cell function is reflected by seminal inhibin B, which has shown predictive value in some cohorts [68,69], whereas seminal AMH did not predict extraction outcome—a useful negative result that delimits the panel [70]. Collectively, these proteins are mechanistically plausible and several are assay-tractable, but most remain at the discovery stage with limited orthogonal or external validation. Systemic and seminal inflammatory indices, such as the systemic immune-inflammation index, have also been associated with retrieval, consistent with a contribution of the inflammatory-oxidative milieu to spermatogenic failure [71].

### 4.3. Metabolomics and Lipidomics

Metabolomic and lipidomic profiling adds an orthogonal molecular layer that integrates the net biochemical activity of the seminiferous epithelium and accessory glands. Untargeted gas chromatography–mass spectrometry distinguished TESE-positive from TESE-negative NOA men and fertile controls with ROC values above 0.88 [72], and broader metabolomic studies have separated azoospermia subtypes and histological patterns. Vibrational and resonance spectroscopy provide label-free fingerprints: combined Raman and nuclear-magnetic-resonance spectral features with reactive-oxygen-species measures separated retrieval-positive from retrieval-negative seminal plasma with ROC AUCs of ~0.81–0.86 [73]. Etiology-specific metabolomics is also emerging; in cryptorchidism-related azoospermia, seminal-plasma metabolic signatures distinguished successful from failed micro-TESE where routine clinical parameters did not [74]. As with the proteomic data, these signatures derive from small, single-center cohorts and lack external validation, and they are particularly sensitive to the accessory-gland and pre-analytical confounders discussed earlier. Comparable proteomic and metabolomic discovery strategies are being applied across male-infertility phenotypes, including varicocele-associated infertility, underscoring both the promise and the standardization challenges of seminal-omics approaches [75].

### 4.4. Oxidative Stress and Redox Biomarkers

A complementary molecular axis is the seminal redox state. Excessive reactive oxygen species (ROS) damage germ cells and sperm DNA and contribute to spermatogenic failure, and seminal ROS and oxidation–reduction potential are measurable non-invasively [76]. Consistent with this biology, spectroscopic models combining vibrational and resonance features with ROS measures showed moderate discrimination of retrieval outcome (Section 4.3), although the ROS component itself did not reach statistical significance in that small proof-of-concept study; redox markers may nonetheless serve as a feasible, low-cost component of a future multi-analyte panel. The individual seminal protein and metabolite biomarkers discussed in this section are summarized in Table 2.

## 5. From Molecules to Models: Stage Mapping, Assay-Readiness and AI-Ready Integration

### 5.1. Mapping Analytes to Spermatogenic Stage

**Figure 2:**
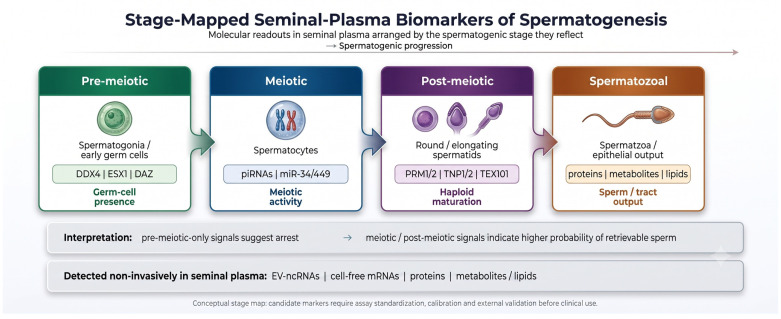
Stage-mapped seminal-plasma biomarkers of spermatogenesis. Molecular species, non-invasively detectable in seminal plasma, are mapped to the germ-cell stage they reflect, from pre-meiotic spermatogonia (e.g., DDX4, ESX1) through meiotic spermatocytes (pachytene PIWI-interacting RNAs, miR-34/449) to post-meiotic spermatids (protamines, TNP1, SPEM1, ZMYND15, TEX101, LDHC) and spermatozoa. Detection of meiotic and post-meiotic/haploid markers indicates completion of meiosis and a higher probability of retrievable testicular spermatozoa, whereas pre-meiotic-only signals suggest spermatogenic arrest.

A practical way to organize this molecular landscape is to map each analyte to the spermatogenic stage it reflects, together with its matrix of origin, reported discrimination and analytical maturity (Figure 2 and Table 3). The proof of concept that combining analytes of distinct biological origin outperforms any single marker is the TEX101 + ECM1 pair, which couples a germ-cell production signal with an epididymal transport signal [60,61].

Figure 2 should be read from left to right as a developmental stage map of spermatogenesis, not as a ranking of individual biomarkers. Pre-meiotic transcripts (*DDX4*, *ESX1*, *DAZ*) indicate germ-cell presence but do not prove completion of meiosis. Meiotic small-RNA signals—pachytene piRNAs and miR-34/449-family members—mark progression through meiotic programs. Post-meiotic/haploid markers (*PRM1*/*2*, *TNP1*/*2*, *SPEM1*, *ZMYND15*, and the proteins TEX101 and LDHC) are more directly tied to haploid differentiation and thus to the presence of retrievable spermatozoa. The map is therefore directional: pre-meiotic-only signals are compatible with maturation arrest, whereas meiotic and post-meiotic markers point to completed meiosis and a higher retrieval probability. Because spermatozoal and accessory-gland outputs add non-testicular signal, the figure is best used to assemble a stage-spanning, multi-analyte panel rather than to interpret any single marker in isolation.

### 5.2. Assay-Readiness and Standardization

Translation is presently constrained less by the shortage of candidate markers than by analytical maturity. Discovery proteomes and RNA-sequencing datasets overlap poorly across laboratories; EV isolation methods (ultracentrifugation, precipitation, size-exclusion chromatography) recover different vesicle subpopulations; RNA normalization strategies are heterogeneous and frequently rely on reference genes of uncertain stability in seminal plasma; and reference ranges, quality-control materials and inter-laboratory harmonization are largely absent. Specific pre-analytical variables that require standardization before any seminal signature can become an AI-ready feature include the time and temperature from ejaculation to processing [78,79], the abstinence interval, centrifugation and extracellular-vesicle isolation protocol, RNA and protein normalization, storage and freeze–thaw cycles, and leukocytospermia or inflammatory contamination [23]. Moreover, few seminal extracellular-vesicle studies report characterization compliant with the Minimal Information for Studies of Extracellular Vesicles (MISEV) reporting framework [80,81], which further limits cross-study comparability. The migration from discovery mass spectrometry to a targeted, antibody-validated assay—as achieved for TEX101 through a dedicated sample pre-treatment and immunoassay protocol—remains the exception rather than the rule [61]. Until pre-analytical and analytical standardization is achieved, cross-study comparison and panel construction will remain unreliable, and most candidates will stay pre-clinical. Ejaculatory abstinence is a further source of variability, affecting both conventional semen parameters and sperm DNA integrity [82,83,84]; a fixed abstinence interval, consistent with the WHO manual [85], should therefore be specified [23]. The microbiological and inflammatory status of the sample is likewise relevant, since leukocytospermia and bacteriospermia modify sperm DNA integrity and the seminal cytokine and microRNA profile [40,86,87,88,89]; leukocyte counts and screening for genital-tract infection should accordingly be recorded.

### 5.3. Prediction Models and the Discovery–Deployment Divide

A striking feature of the prediction literature is that the best-performing models for sperm retrieval are built almost entirely on clinical, hormonal, histological, imaging and genetic variables rather than molecular omics. Prediction modeling has matured over two decades, and its trajectory is instructive. Early artificial neural networks (ANNs) trained on hormonal and clinical inputs reported good apparent accuracy—an ANN incorporating leptin outperformed FSH alone (AUC 0.83 vs. 0.63), and logistic equations reached AUCs of ~0.83 in idiopathic NOA [90,91,92]. Head-to-head comparisons were sobering: in micro-TESE specifically, an ANN, a nomogram and logistic regression all performed only modestly (ANN test AUC ~0.64), showing that high training performance need not transfer [93]. The clearest demonstration of optimism is external validation: a nationwide TESE model retained an AUC of only ~0.65 on independent data despite acceptable development performance [94].

Contemporary clinical prediction models now include large multicenter nomograms and internally validated single-center models, but their discrimination remains heterogeneous. Examples include a 3093-man multicenter nomogram with moderate discrimination (AUC 0.71), in which only testicular volume and age remained independent predictors on multivariate analysis [95], a six-center micro-TESE nomogram with C-index 0.75 [96], and single-center models reporting AUCs from approximately 0.72 to 0.88 with increasing use of calibration and decision-curve analysis [97,98,99]. The largest and most rigorous example trained eight algorithms in more than 2800 NOA men, with extreme gradient boosting (XGBoost) achieving the highest mean AUC (~0.92) and an external-validation AUC of ~0.83, deployed as an online calculator [100]. A comparable machine-learning analysis of salvage (secondary) micro-TESE in 503 men likewise identified extreme gradient boosting as the best-performing algorithm, again drawing only on clinical and laboratory inputs—body mass index, prior extraction site, luteinizing hormone and semen volume [101]. Imaging predictors (testicular ultrasonic microvascular density, shear-wave elastography, diffusion-tensor imaging, seminiferous-tubule caliber) and etiology-specific models for Klinefelter syndrome, cryptorchidism and post-chemotherapy azoospermia add granularity for defined subgroups [102,103,104,105]. Yet across this entire literature the predictor sets remain clinical, hormonal, histological, imaging or genetic; seminal-plasma omics features are essentially absent, so even the strongest models are comparators to, rather than realizations of, an integrated molecular–AI tool. To date no validated model has combined seminal-plasma molecular markers with clinical, hormonal and genetic predictors, so the incremental value of adding molecular features remains an untested hypothesis rather than a demonstrated gain. This is the central discovery–deployment divide. The conventional and histology-dependent predictor literature—including the limited preoperative value of diagnostic testicular biopsy—has been critically reviewed [8,106]. Genetic and endocrine subtyping further refine prediction: Y-chromosome AZFc-deletion architecture influences retrieval, with gr/gr deletions showing lower rates than b2/b4 [107], and composite endocrine indices such as the serum testosterone-to-luteinizing-hormone ratio outperform single hormones in selected cohorts [108]. A related methodological pitfall is predictor timing: models that incorporate testicular histology, Johnsen score or other intra- or post-procedural information can appear highly accurate yet are prone to information leakage and circularity, because such predictors are not genuinely available before surgery; rigorous studies should therefore separate truly preoperative predictors from procedure-dependent ones.

### 5.4. Why Discrimination Is Not Deployment

**Figure 3:**
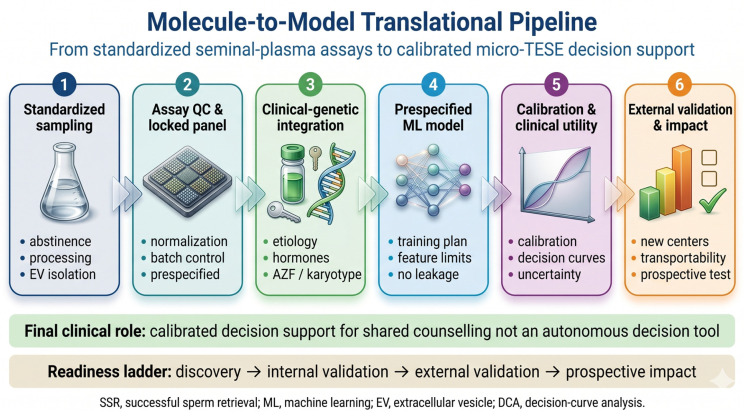
Molecule-to-model translational pipeline. Proposed workflow from standardized seminal sampling and extracellular-vesicle isolation, through RNA/protein extraction, biomarker quantification (RT-qPCR/ddPCR, ELISA or targeted mass spectrometry), normalization and assay quality control, to a locked, stage-mapped biomarker panel integrated with clinical, hormonal and genetic predictors in a prespecified machine-learning model evaluated for calibration, decision-curve net benefit and internal/external validation (TRIPOD + AI/PROBAST + AI) and, ultimately, prospective impact testing supporting shared counseling. Current seminal-plasma and AI/ML models remain at the discovery–internal-validation stage, and any future tool is intended as a decision-support adjunct, not an autonomous decision tool.

Even where discrimination is high, it is insufficient for clinical use. Discrimination (the AUC or C-index) measures only the rank-ordering of patients; it is silent about calibration, the agreement between predicted and observed probabilities. A miscalibrated model with an impressive AUC can convert a true 5% probability of retrieval into a predicted 30%, or vice versa, directly distorting the counseling that the model is meant to support. Clinical usefulness additionally requires decision-curve analysis to demonstrate net benefit across the threshold probabilities relevant to couples and surgeons, and trustworthy performance requires independent external validation across centers and etiologies, ideally with prospective impact assessment. These requirements are now codified in the TRIPOD + AI reporting standard and the PROBAST + AI risk-of-bias framework [109], yet most retrieval-prediction studies still report discrimination alone [110]. An explicit molecule-to-model pipeline is therefore advocated: a small, locked, stage-mapped panel of seminal-plasma analytes—combining, for example, an EV non-coding-RNA signal, a post-meiotic cfs-mRNA marker and the TEX101/ECM1 protein readout—integrated with routinely available clinical and genetic predictors, developed under a prespecified modeling plan, and evaluated for calibration, net benefit and external transportability before any clinical deployment (Figure 3). It is useful to grade prediction tools along a readiness ladder—from discovery-only, through internal and temporal validation, to external validation and, ultimately, prospective impact testing—and on this scale essentially all current seminal-plasma and AI/ML models for sperm-retrieval prediction remain at the discovery or internal-validation stage.

## 6. Challenges and Future Directions

Three priorities follow from this analysis. First, biological and analytical standardization: the field needs consensus on abstinence interval, EV-isolation method, RNA/protein normalization, and minimum reporting, so that candidate markers can be compared and combined across centers; reference materials and external quality assessment will be essential. Second, study design: prospective, multicenter cohorts should collect standardized preoperative clinical, hormonal, genetic and seminal-molecular data before micro-TESE, with biomarker panels locked prior to analysis and models prespecified, enabling unbiased estimation of incremental value over routine predictors. Third, evaluation: discrimination must be reported together with calibration, decision-curve analysis and independent external validation, accompanied by transparent, reproducible model objects. Methodologically, single-cell and spatial transcriptomics of the NOA testis can refine which germ-cell transcripts are most informative, multi-omic integration (EV-RNA, protein and metabolite) can capture complementary signals, and explainable machine-learning methods can keep such models interpretable for shared decision-making. Throughout, an ethical constraint must hold: any future tool should augment counseling and must not be used to deny micro-TESE to an individual patient on the basis of an unvalidated probability, given that some couples will reasonably choose surgery even at low predicted odds.

Etiology-specific and potentially modifiable factors also warrant integration. A 2025 systematic review and meta-analysis of controlled studies found that varicocele repair in NOA men with clinical varicocele significantly increased the odds of surgical sperm retrieval (OR 2.17, 95% CI 1.17–4.01) and of sperm appearing in the ejaculate (OR 7.8, 95% CI 3.59–16.94) [111], and varicocele grade and testicular histopathology influence these outcomes [112]. Models should likewise distinguish reversible endocrine NOA from primary testicular failure, because gonadotropin therapy can induce ejaculated or retrievable spermatozoa in hypogonadotropic hypogonadism [113].

A notable evidence gap should guide priorities: although meta-analyses exist for hormonal predictors, surgical technique and Klinefelter syndrome, no meta-analysis has yet pooled the diagnostic accuracy of seminal non-coding-RNA biomarkers or the performance of AI/ML retrieval models, reflecting the heterogeneity, small samples and absent external validation of those studies. Standardized, harmonized reporting is the prerequisite for such quantitative synthesis. Multi-omic and single-cell frameworks, combined with standardized assays, offer a path toward integrating germline, niche and epigenetic data into validated, externally tested prediction.

Because NOA is etiologically heterogeneous, seminal-plasma biomarkers should not be interpreted independently of genetic architecture. Karyotype, azoospermia-factor (AZF) status and emerging monogenic causes define biological subgroups with distinct probabilities of residual spermatogenesis and sperm retrieval [77,114,115]. Future seminal-plasma biomarker models should therefore be etiology-aware rather than universal.

Adjacent decision-support technologies are advancing in parallel, including artificial-intelligence-assisted sperm detection in surgical samples, automated interpretation of touch-print smear cytology, and deep-learning identification of sperm-containing seminiferous tubules [116,117,118]. These tools may streamline laboratory, cytological and intraoperative workflows, but they operate during or after surgery and do not replace the need for a preoperative, non-invasive molecular readout of residual spermatogenesis.

## 7. Conclusions

Seminal plasma is a biologically coherent molecular liquid biopsy of spermatogenesis. Extracellular vesicles, non-coding RNAs, cell-free germ-cell transcripts, testis-enriched proteins and metabolites carry stage-specific information about residual sperm production in NOA, and the directional anatomy of the blood–testis barrier confers specificity by channeling germ-cell molecules toward the ejaculate rather than the blood. The molecular evidence is rich and mechanistically plausible, and individual markers—most maturely the TEX101/ECM1 protein pair—approach analytical readiness. Yet no validated, calibrated, externally tested tool currently integrates seminal-plasma multi-omics with clinical and genetic predictors for preoperative micro-TESE decision support, and the best available prediction models scarcely use molecular inputs at all. Bridging this molecule-to-model gap—through standardized assays, biologically rational stage-mapped multi-analyte panels, and prediction-model development that satisfies modern reporting and validation standards—is the decisive step toward a clinically useful, non-invasive prediction of sperm retrieval in non-obstructive azoospermia. At present, however, seminal plasma remains a research platform rather than a clinically deployable test, and prospective impact testing is required before clinical use.

## Figures and Tables

**Table 1:** Seminal RNA biomarkers evaluated for sperm-retrieval prediction in non-obstructive azoospermia.

Biomarker	Class/Matrix	Reflects (Stage/Function)	Key Reported Findings	Ref.
miR-31-5p	miRNA; seminal EV/plasma	Germ-cell presence; SSC proliferation/apoptosis (JAZF1, cyclin A2)	NOA vs. OA; predicts testicular sperm; AUC ≈ 0.93 (with FSH)	[23,33,34,35]
miR-27a	miRNA; seminal	Represses CRISP2 (motility/acrosome); ↑ in NOA (↓ KDM3A)	Differentially expressed; circRNA–miRNA axis	[37,38,39]
miR-146a-5p	miRNA; seminal	Inflammation-associated	Altered in infertile men	[37,40]
miR-34b/c, miR-449	miRNA cluster; testis	Meiotic progression; histone-to-protamine; loss → arrest	Mechanistic, testis-enriched	[32]
9-lncRNA EV panel	lncRNA; seminal EV	Stage-specific germ-cell programs	Training/validation AUC 0.99/0.96	[41]
exLncRNA ratio pairs	lncRNA; seminal EV	Ratio-based germ-cell signal	Classifies retrieval above threshold	[42]
TUG1, CDKN2B-AS1, H19 (+Linc-ROR, MALAT1, MIAT, GAS5)	lncRNA; seminal	Chromatin/cancer-associated	TUG1 AUC 0.94; CDKN2B-AS1/H19 AUC 0.90 (diagnostic)	[43]
3-circRNA panel	circRNA; seminal plasma	Testis-derived; meiotic/post-meiotic	Individual AUC 0.89–0.93; combined LASSO 0.96	[44]
pir-61927	piRNA; seminal EV	Pachytene/post-meiotic germ cells	Training/validation AUC 0.82/0.83	[49]
tRF-Val-AAC-010 (+tRF-Pro-AGG-003)	tRF; seminal EV	Germ-cell/epididymal	Origin AUC ≈ 0.96; retrieval AUC 0.89 (72%/91%)	[50]
*DDX4* (VASA)	cfs-mRNA; seminal	Pre-meiotic germ cells	Depleted in SCO	[52]
*DAZ*/*DAZL*	cfs-mRNA; seminal	Pre-meiotic germ cells	Stage marker	[52]
*ESX1*	cfs-mRNA; seminal	Pre-/post-meiotic	~80% NOA detectable; retrieval ~84% sensitivity; ↓ in NOA	[54,55,56]
*PRM1*/*PRM2*	cfs-mRNA; seminal	Post-meiotic (haploid)	Completion of meiosis; *PRM1* ↓ in severe impairment, correlates with ICSI pregnancy	[53,56,58]
*TNP1*/*TNP2*	cfs-mRNA; seminal	Post-meiotic (haploid)	Completion of meiosis	[53,56]
*SPEM1*	cfs-mRNA; seminal	Post-meiotic (haploid)	AUC 0.91	[53]
*ZMYND15*	cfs-mRNA; seminal	Spermatid repressor (gates *PRM1*/*TNP1*/*SPEM1*)	↓ in NOA; predictive	[53,56]
*BOLL*, *AKAP1*, *TCP11*, *SETX*	cfs-mRNA; seminal	Completion of meiosis	Multi-stage screen candidates	[57]

AUC, area under the receiver-operating-characteristic curve; cfs-mRNA, cell-free seminal mRNA; EV, extracellular vesicle; FSH, follicle-stimulating hormone; ICSI, intracytoplasmic sperm injection; LASSO, least absolute shrinkage and selection operator; NOA/OA, non-obstructive/obstructive azoospermia; SCO, Sertoli-cell-only; SSC, spermatogonial stem cell.

**Table 2:** Seminal protein and metabolite biomarkers evaluated for sperm-retrieval prediction in non-obstructive azoospermia.

Biomarker	Class/Matrix	Reflects (Stage/Function)	Key Reported Finding	Ref.
TEX101	Protein; seminal plasma	Late spermatogenesis (spermatocytes/spermatids)	NOA vs. OA 81% sens at 100% spec; ≥0.6 ng/mL → retrieval 73% sens, 64% spec (n = 805)	[60,61]
ECM1	Protein; seminal plasma	Epididymal/ductal patency	Pairs with TEX101 (production vs. transport)	[60,61]
LDHC, PGK2	Protein (glycolytic); seminal	Germ-cell/testis-enriched	↓ LDHC in NOA/SCO; PGK2 ↑ in successful retrieval (cutoff 136.3 pg/mL)	[62,63]
HSPA2, HSPA4L	Protein (chaperones); seminal	Germ-cell	↓ HSPA2 in NOA/SCO	[62]
DPEP3	Protein (metallopeptidase); seminal	Testis-restricted	Discovery candidate	[62]
ACR (acrosin)	Protein; seminal	Acrosomal	↑ in successful retrieval (cutoff 21.75 mIU/mL)	[63]
LGALS3BP	Protein; seminal	Germ-cell/secreted	Predictor of successful extraction	[64]
Galectin-1	Protein; seminal	—	Inverse with Johnsen score; diagnostic AUC 0.86	[65]
Survivin	Protein (IAP); seminal	Spermatogenic activity	Declines with spermatogenic failure; proposed retrieval marker	[11,66]
Angiotensin II	Peptide; seminal	—	Lowest in SCO histology	[67]
Inhibin B	Protein; seminal	Sertoli-cell function	Predictive in some cohorts	[68,69]
AMH	Protein; seminal	—	Did not predict extraction (negative result)	[70]
GC-MS metabolomic signature	Metabolomics; seminal	Integrated epithelial activity	TESE-positive vs. -negative; ROC > 0.88	[72]
Raman/NMR + ROS features	Spectroscopy; seminal	Integrated biochemical state	ROC ≈ 0.81–0.86	[73]
Cryptorchidism metabolic signature	Metabolomics; seminal	Etiology-specific	Distinguished successful vs. failed micro-TESE	[74]
Seminal ROS/redox potential	Redox; seminal	Oxidative milieu	Measurable; modest/non-significant alone	[76]

AUC, area under the receiver-operating-characteristic curve; GC-MS, gas chromatography–mass spectrometry; IAP, inhibitor of apoptosis; NMR, nuclear magnetic resonance; NOA/OA, non-obstructive/obstructive azoospermia; ROC, receiver-operating-characteristic; ROS, reactive oxygen species; SCO, Sertoli-cell-only.

**Table 3:** A molecule-to-model overview of seminal-plasma analytes for predicting sperm retrieval in non-obstructive azoospermia.

Analyte Class (Examples)	Matrix	Spermatogenic Stage Reflected	Reported Discrimination	Assay-Readiness	Reference(s)
microRNA (miR-31-5p)	Seminal EV/plasma	Meiotic regulation; germ-cell presence	AUC ≈ 0.9–0.93 (with FSH)	Moderate (qPCR/ddPCR)	[23,33,35]
lncRNA (9-EV panel; exLncRNA pairs)	Seminal EV	Stage-specific germ-cell programs	Training/validation AUC 0.99/0.96	Low–moderate (panel, normalization)	[41,42,43]
circRNA (3-circRNA panel)	Seminal plasma	Testis-derived; meiotic/post-meiotic	Combined AUC 0.96	Low–moderate (stable target)	[44]
piRNA (pir-61927)	Seminal EV	Pachytene/post-meiotic germ cells	AUC 0.82–0.83	Low (discovery)	[49]
tRF (tRF-Val-AAC-010)	Seminal EV	Germ-cell/epididymal	AUC 0.89 (retrieval)	Low (discovery)	[50]
cfs-mRNA, pre-meiotic (*DDX4*, *ESX1*, *DAZ*)	Seminal plasma	Spermatogonia/spermatocytes	*ESX1* ~84% sensitivity	Moderate (RT-qPCR)	[52,54,55]
cfs-mRNA, post-meiotic (*PRM1*/*2*, *TNP1*, *SPEM1*, *ZMYND15*)	Seminal plasma	Haploid spermatids (completion of meiosis)	*SPEM1* AUC 0.91	Moderate (RT-qPCR)	[53,56,58]
Protein pair (TEX101 + ECM1)	Seminal plasma	Late spermatogenesis + ductal patency	81% sens at 100% spec (NOA vs. OA)	High (validated ELISA)	[60,61]
Proteomic panel (LDHC, PGK2, HSPA2, DPEP3)	Seminal plasma	Germ-cell/testis-enriched	Discovery-stage	Low–moderate (targeted MS/ELISA)	[62,63,64]
Metabolomics/spectroscopy	Seminal plasma	Integrated epithelial activity	ROC 0.81–0.95	Low (discovery)	[72,73,74]
Clinical/hormonal (FSH, inhibin B, TV)	Serum/clinical	Sertoli/global testicular function	Inconsistent alone	High (routine)	[7,68,69,70]
Genetic (AZF, NGS gene panels)	Blood/genetic	Etiologic	Subgroup-specific	High (routine where indicated)	[1,6,77]

AUC, area under the receiver operating characteristic curve; cfs-mRNA, cell-free seminal mRNA; ddPCR, droplet digital PCR; ELISA, enzyme-linked immunosorbent assay; EV, extracellular vesicle; FSH, follicle-stimulating hormone; MS, mass spectrometry; NOA/OA, non-obstructive/obstructive azoospermia; RT-qPCR, reverse-transcription quantitative PCR; TV, testicular volume. Reported discrimination values are from heterogeneous, mostly single-center studies and are not directly comparable.

## Data Availability

No new data were created or analyzed in this study. Data sharing is not applicable to this article.

## Abbreviations

The following abbreviations are used in this manuscript:

AMH	anti-Müllerian hormone
AUC	area under the receiver operating characteristic curve
BTB	blood–testis barrier
cfs-mRNA	cell-free seminal mRNA
circRNA	circular RNA
ECM1	extracellular matrix protein 1
ELISA	enzyme-linked immunosorbent assay
ESCRT	endosomal sorting complex required for transport
EV	extracellular vesicle
FSH	follicle-stimulating hormone
ICSI	intracytoplasmic sperm injection
LC-MS/MS	liquid chromatography–tandem mass spectrometry
lncRNA	long non-coding RNA
micro-TESE	microdissection testicular sperm extraction
miRNA	microRNA
NOA	non-obstructive azoospermia
OA	obstructive azoospermia
piRNA	PIWI-interacting RNA
PRM	protamine
TEX101	testis-expressed protein 101
tRF	tRNA-derived fragment
XGBoost	extreme gradient boosting
